# Unilateral Hydrocephalus Due to Lateral Ventricle Colloid Cyst Treated by Neuroendoscopic Approach

**DOI:** 10.7759/cureus.7825

**Published:** 2020-04-25

**Authors:** Clauder Oliveira Ramalho, Amanda Viguini Tolentino Correa, Tiago Hilton Vieira Madeira, Alexandre Nascimento Ottoni, Samuel Tau Zymberg

**Affiliations:** 1 Neurosurgery, Hospital Santa Rita de Cássia, Vitoria, BRA; 2 Neurologia, Faculdade Brasileira Multivix, Vitoria, BRA; 3 Neurosurgery, Universidade Federal de São Paulo, São Paulo, BRA

**Keywords:** colloid cysts, foramen of monro, third ventricle, lateral ventricle

## Abstract

Colloid cysts (CCs) are rare brain tumors that cause nonspecific neurological signs associated with acute or chronic increased intracranial pressure. They are usually located in the third ventricle and rarely in the lateral ventricle. This is a report of an unusual case of CC located in the lateral ventricle. A 36-year-old male patient presented a story of progressive holocranial headache that would get worse with head mobilization and cough. Radiological analysis demonstrated enlargement of the right lateral ventricle due to a cyst blocking the right foramen of Monro. The patient underwent endoscopic neurosurgery and the cyst was totally resected. Histological evaluation diagnosed a CC. Postoperative images showed no cyst remaining and normalized ventricular size. The patient evolved with total improvement of the symptoms. Literature review shows that it is a very uncommon entity. Lateral ventricle CCs as a cause for unilateral hydrocephalus is a very rare entity. Neuroendoscopic approach is a first-line treatment option for this condition.

## Introduction

Colloid cysts (CCs) are rare congenital lesions, representing only 0.5%-1.0% of primary brain tumors, and comprises approximately 15%-20% of intraventricular lesions [1]. Usually located in the roof and anterior part of the third ventricle, they are extremely rare outside this area [2]. We present a patient with progressive headache due to a CC in the right lateral ventricle, obstructing the foramen of Monro and causing unilateral ventricle dilatation, which is rarely reported [3]. The clinical condition, the imaging findings, the endoscopic neurosurgical procedure, and the review of the literature are presented as well.

## Case presentation

A 36-year-old male patient, in current treatment for rheumatoid arthritis, presented for neurosurgical evaluation due to progressive holocranial headache that would get worse with head mobilization and cough. The neurological examination was uneventful.

**Figure 1:**
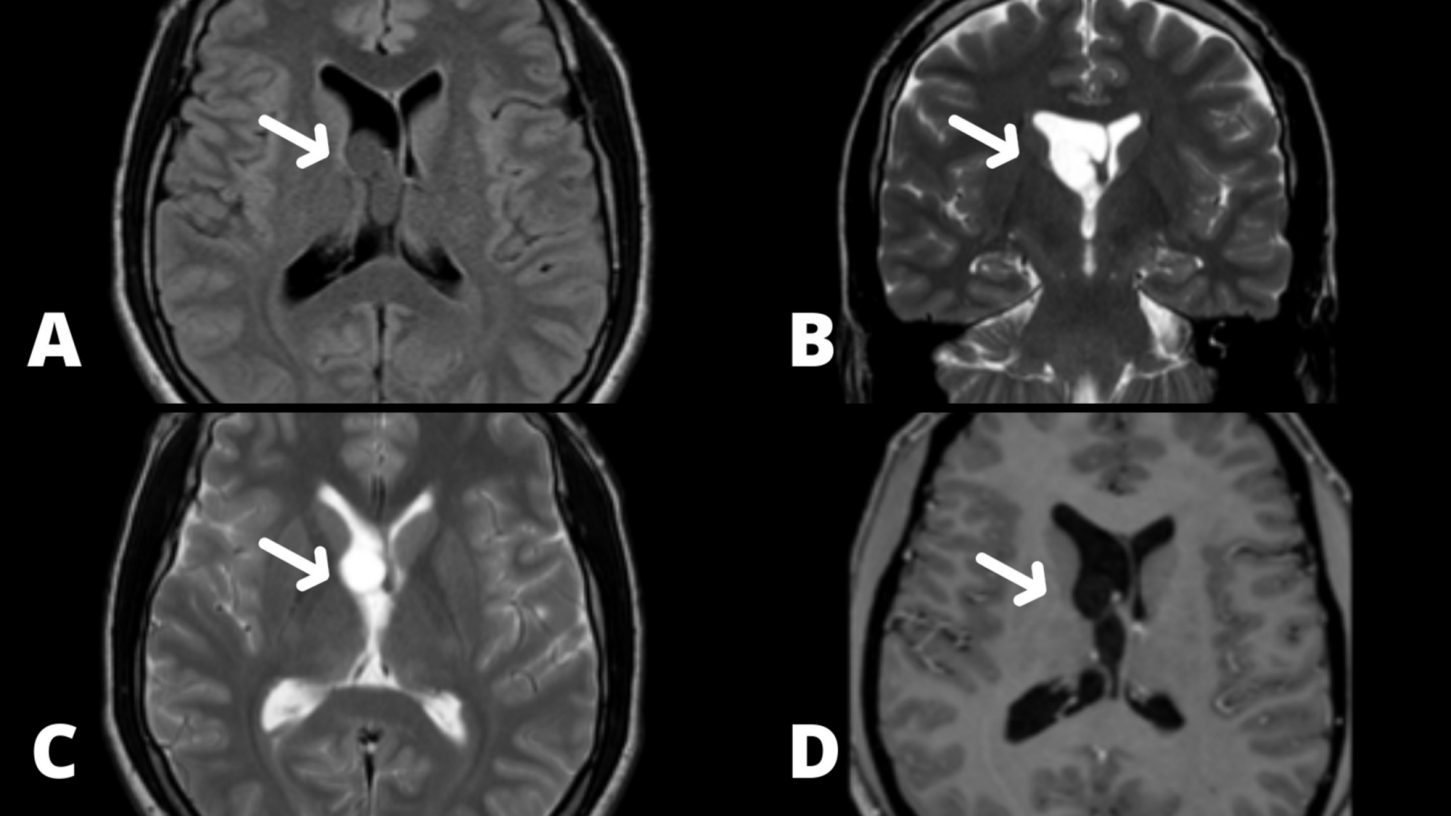
Preoperative axial MRI scan. The images A-D show an enlargment of the right lateral ventricle due to a cyst blocking the right foramen of Monro.

He was previously evaluated at another service and was already diagnosed with right lateral ventricle cyst obstructing the foramen of Monro with minimum ventricular dilatation. Because of the lightness of the symptoms at that moment, medical treatment was prescribed by a clinical neurologist. However, due to worsening of the symptoms, new radiological evaluation was performed. It demonstrated enlargement of the cyst and right lateral ventricle dilatation (Figure 1). Therefore, he was sent to neurosurgical evaluation and neuroendoscopic treatment was proposed. 

Right ventricular endoscopic approach was performed. The patient was positioned in supine, with head in neutral position, flexed in 30 degrees. The skin was incised in a position to permit a right trepanation 3 cm lateral to the midline and 2 cm anterior to the coronal suture, as preoperative study showed it was the best initial point to a route passing through the axis of the cyst to the foramen of Monro.

**Figure 2:**
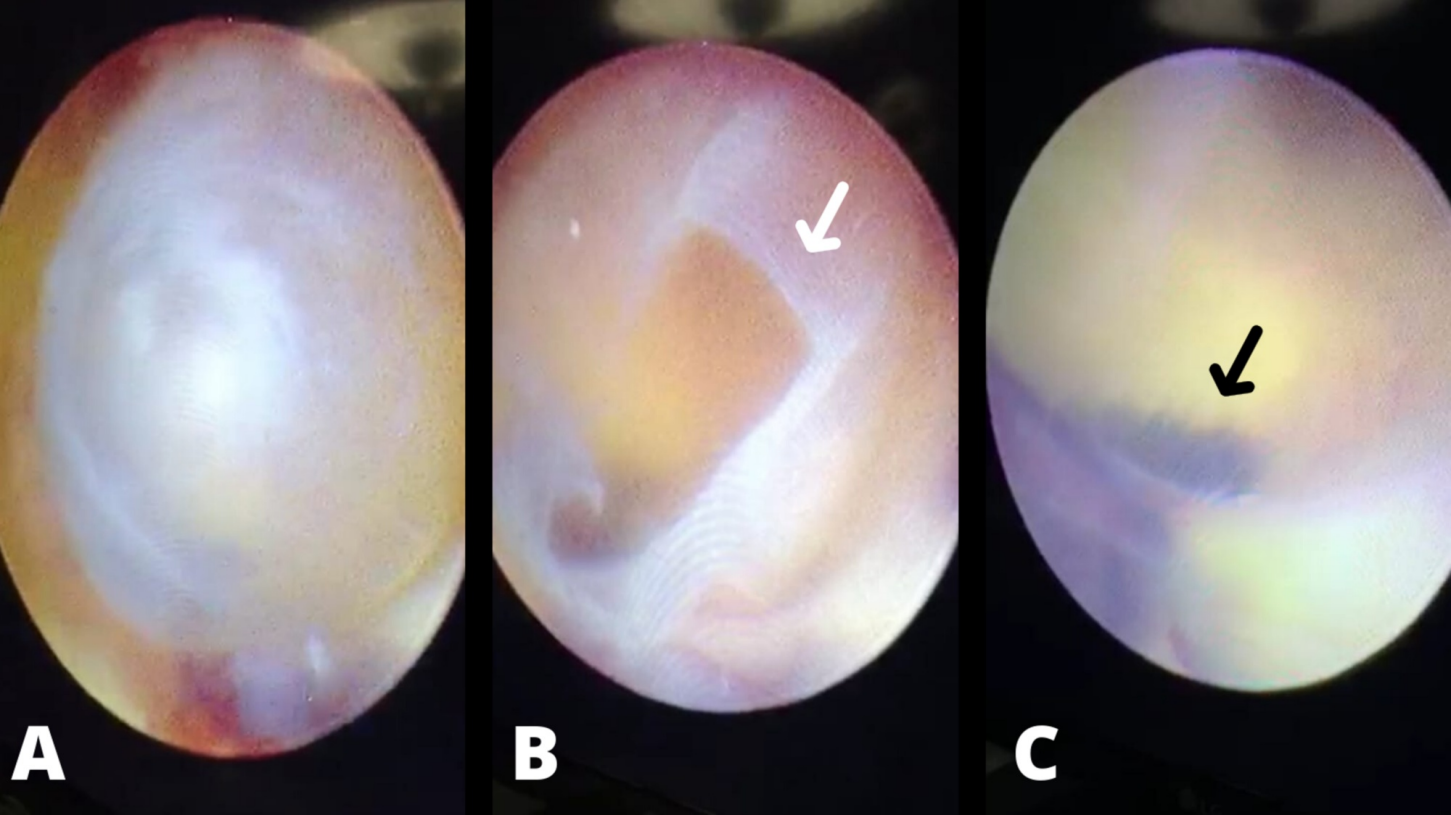
Intraoperative images. Intraoperative images showing the cyst obstructing the foramen of Monro. (A) Shows the cyst before its opening. (B) Shows the cyst after its opening (white arrow). (C) Shows the cyst obstructing the foramen of Monro (black arrow).

**Figure 3:**
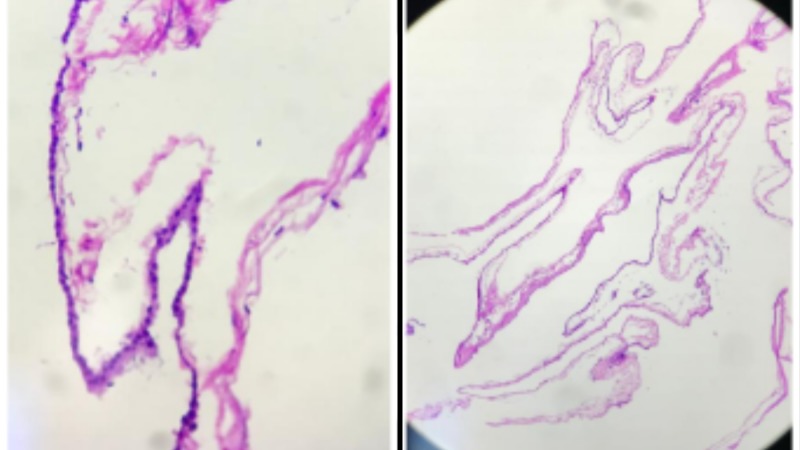
Histological evaluation of the cyst. Histologic evaluation of the cyst showing an outer fibrous layer and inner epithelium of ciliated or mucin-producing cells.

The right ventricle puncture was achieved and the endoscopic set was inserted. A large cyst obstructing the foramen of Monro was easily seen in the right ventricle (Figure 2). After its opening with micro-scissor, white colloid fluid was identified inside. The cyst was totally drained with 4F Fogarty catheter and light syringe aspiration. After removal of the capsule with micro-scissor and forceps and coagulation of the few remnant tissues adherent to blood vessels, the foramen of Monro was cleared unblocked. There was no need of pellucidum septostomy. Tissue and cyst content samples were sent to pathological evaluation, which showed CC as the result (Figure 3).

**Figure 4:**
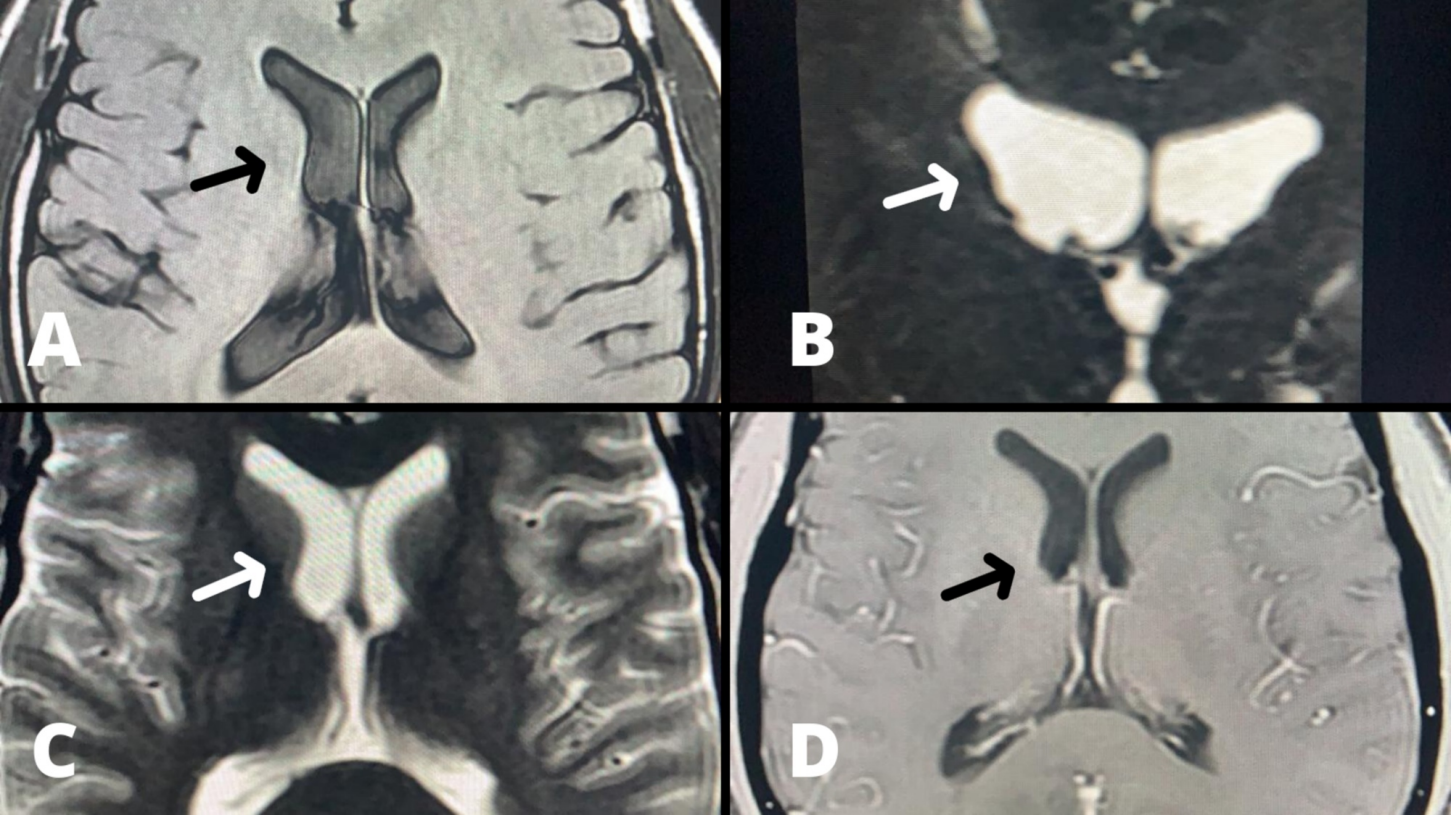
Postoperative axial MRI scan. The images A-D show normalized ventricular size after neuroendoscopic approach.

The ICU length of stay was of one day and the length of hospitalization was of three days. The patient had totally improved and the two-month postoperative images showed normalized ventricular sizes and no cyst remaining (Figure 4).

## Discussion

The CC, also known as neuroepithelial cyst, was first described by Wallmann in 1858 and was first resected by Dandy in 1921. It is composed of an outer fibrous layer and an inner epithelium of ciliated or mucin-producing cells [4].

It represents 0.5%-1% of encephalic tumors and 15%-20% of intra-ventricle tumors, classically in the superior and anterior part of the third ventricle, causing dislocation of the fornices [2, 5]. In less than 1% of the cases, it occurs in other locations, as the lateral ventricles, as seen in the presented case [2]. Unilateral hydrocephalus due to foramen of Monro obstruction by CCs is a very rare condition [3].

Barbagallo et al. reviewed out-of-third ventricle CCs. They performed a Medline web-search, using “colloid cysts”, “lateral ventricle colloid cysts”, “fourth ventricle colloid cysts”, “brain colloid cyst”, and “spinal colloid cysts” as keywords. The literature review revealed only 16 papers reported between 1952 and 2016, evidencing the rarity of this entity. They suggest that in cases of recurrent headache with unusual features and lacking signs of neurological deficits, a rare neurosurgical condition such as an “ectopic” colloid cyst should be included in the differential diagnosis [6].

Uno et al. described a suprasellar CC resected through an endoscopic endonasal transsphenoidal approach. It was diagnosed in a six-year-old boy with mild headache. Although as the initial conservative treatment, when he was 17 years old a resection was performed due to the great enlargement of the lesion and to obtain definitive histological diagnosis. The postoperative course was uneventful [7].

Arooj et al. performed a suboccipital craniotomy to resect a focal well-defined rounded cystic lesion along the fourth ventricle, showing subtle peripheral rim enhancement in a young female with significant hydrocephalus. The histopathology was consistent with a CC. CC is rarely found in infratentorial location [8].

Rizk and Bettag showed a case of a 40-year-old female patient who presented with three intraventricular cystic lesions: one cyst in the typical localization in the anterior rostral third ventricle, another cyst behind it in the same ventricle, and a larger bulging cyst in the right lateral ventricle. The cysts had different MRI signal characteristics and showed different consistencies during the surgical approach [9].

The CC typically causes nonfocal neurological signs, associated with acute or chronic increased intracranial pressure. Headache is the most common symptom. Sudden death is a possibility [10]. These symptoms are usually presented in Bruns syndrome.

CT images show the density of the cyst and it may correlate with its hydration state. However, CCs diversify greatly on MRI scans. The signal characteristics vary according to the cyst composition. Usually, T1-weighted signal intensity is correlated with cholesterol concentration. The T2-weighted signal intensity is variable.

The CC management is controversial regarding the decision whether treatment is really necessary, as long as some cysts are incidental. Witt Hamer et al. described 34% risk of acute deterioration and overall mortality of 12% [11]. Resection of the cyst may be indicated in symptomatic patients, when enlargement of the cyst occurs or when hydrocephalus is diagnosed due to cyst obstruction [10, 12-13].

Although there are some papers describing other surgical approaches, as stereotactic aspiration, the most popular ones are neuroendoscopic and microsurgical approaches [14-17]. The neuroendoscopic approach is considered a first-line treatment in cases of ventricular dilatation because it is safe and effective, although there is a higher risk of partial resection, but it varies with neurosurgeon experience. The complications incidence is much lower when compared to microsurgical approach [18-20].

The patient of this paper underwent surgery because he presented symptomatic unilateral hydrocephalus due to foramen of Monro obstruction by a colloid cyst in the lateral ventricle. The neuroendoscopic approach was elected as the treatment because of the ventricular dilatation, its location near the foramen of Monro, and the team experience. The surgery was uneventful, the patient had improved, and postoperative radiological findings indicated no cyst remaining and normal ventricular size.

## Conclusions

Unilateral hydrocephalus because of a colloid cyst in the lateral ventricle is a very rare entity. The surgical approach for CC may be indicated when it is symptomatic. Neuroendoscopy is a first-line treatment for these lesions, with low morbidity and short hospital stay.
